# North Korea Must be Global Scale Cohort, Not a Galapagos in the Medical Research Field

**DOI:** 10.31557/APJCP.2019.20.9.2789

**Published:** 2019

**Authors:** Duck Yeong Ro, Dae Hee Kim, Sae Hyun Park

**Affiliations:** 1 *Department of Obstetrics and Gynecology,*; 2 *Department of Emergency Medicine, Incheon St. Mary's Hospital, College of Medicine, The Catholic University of Korea, Seoul, *; 3 *Department of Obstetrics and Gynecology, Incheon-sarang Hospital, Incheon, Republic of Korea. *

**Keywords:** Research trends, gynecologic cancer, North Korea, socioeconomic disparity, genetic identity

## Abstract

**Objective:**

This study aimed to compare the research trend regarding gynecologic malignancies in North Korean medical journal and South Korean medical journal.

**Methods:**

Articles published in the journal of “Pediatrics, Obstetrics, and Gynecology” in North Korea and “Obstetrics & Gynecology Science” in South Korea from 2006 to 2016 were analyzed by using frequency analysis. Studies on gynecologic malignancies were classified by international classification of disease (ICD-10).

**Results:**

Out of 3361 reviewed articles, 116 articles published in North Korean journal and 519 articles published in South Korean medical journal were classified as gynecologic oncology. We found a distinct difference between North and South Korean medical journals regarding research trends on gynecologic oncology. The proportions of gestational trophoblastic disease, cervical cancer, and anogenital warts were higher in North Korean medical journal, but proportions of ovarian cancer, fallopian tube cancer, peritoneal cancer, corpus uterine cancer, and vulvar cancer were higher in South Korean medical journal.

**Conclusion:**

This study enforced an analysis of research trends on gynecologic malignancies in North Korean and South Korea medical journals, and a distinct difference was observed in this regard. In the future, grand scale cohort study in the genetic identical two Korean population is needed for research of environmental effect on gynecologic cancer.

## Introduction

The long division between North and South Korea has resulted in an increase in the socioeconomic disparities and heterogeneity between two societies, creating a notable difference between two Koreas in terms of quality of health care services (Park et al., 2018a). 

North Korea may be the Galapagos of the medical research field. This country is isolated, thus any kind of disclosure of healthcare conditions internally or externally is avoided. Isolation of North Korea versus accessibility of South Korea may be s prime research area for evaluation of environmental influence on various diseases based on genetic identity. North Korea represent nuclear crisis, whereas South Korea represent BTS – the world pop star! The Korean peninsula may be a unique cohort on a global scale (Kim et al., 2016).

However, conducting research on North Korea is difficult because of the lack of primary data sources. Difficulties in securing credible data caused many South Korean researchers make various attempts for intelligence collection; for example, conducting face-to-face talk with North Korean defectors. Nevertheless, generalization of those research data is not easy. Hence, some studies investigated medical documents of North Korean (Park et al., 2018a). Medical journal is a useful measure for exchanging information about a population healthcare status. Furthermore, it is the least political in the extremely political North Korean society. Through the analysis of medical journals, we can make conjecture about current status of healthcare by getting information on the academic interests within a society, and we can also understand the level of medical development by obtaining information on technological equipment across different studies (Ha and Lee, 2018).

**Table 1:** Comparison of Proportion of Articles Classified by Subspecialty between Two Korean Journals

	North Korean journal	South Korean journal
Subspeciallty	n (%)	n (%)
Maternal-fetal medicine	381 (37)	615 (35)
General gynecology	255 (24)	378 (21)
Reproductive endocrinology	171 (16)	236 (13)
Gynecologic oncology	116 (11)	519 (29)
Breast	114 (11)	5 (0)
Not classifed	5 (0)	7 (0)
Summary	1042 (100)	1760 (100)

**Table 2:** Classification of Gynecologic Malignant Diseases which is the Main Research Subject by ICD-10

Gynecologic oncologic disease	North Korea n (%)	South Korea n (%)
Gestational trophoblastic disease	45 (29)	13 (2)
Cervix malignancy	39 (25)	137 (22)
Dysplasia of cervix uteri	7 (4)	28 (5)
Ovary, fallopian tube, peritoneal malignancy	25 (16)	164 (27)
Corpus uteri malignancy	6 (4)	75 (12)
Vulva malignancy	0 (0)	19 (3)
Vagina malignancy	2 (1)	9 (1)
Benign neoplasm of ovary	3 (2)	67 (11)
Anogenital (venereal) warts	6 (4)	0 (0)
Not classified	10 (6)	46 (8)
etc	13 (8)	51 (8)
Summary	156 (100)	609 (100)

**Figure. 1:**
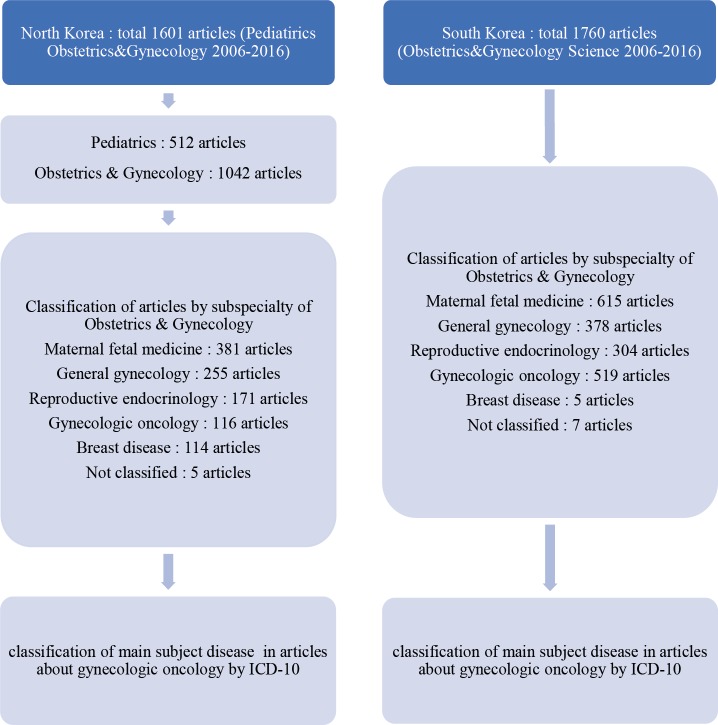
The Journal of "*Pediatrics, Obstetrics, and Gynecology*" Published in North Korea and the Journal of "*Obstetrics and Gynecology Science*" published in South Korea were Selected for this Study, of which Articles were Classified According to Subspecialty, and the Main Research Subject Disease of Articles about Gynecologic Cancer was Classified by ICD-10

**Figure 2:**
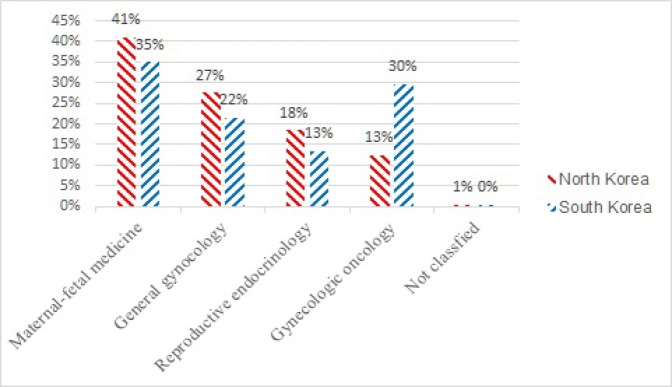
Adjusted Comparison (by Omitting Breast Cancer Subspecialty) of Proportion of Subspecialty-Specific Articles between Two Koreas Showed Lower Proportion of Gynecologic Oncology in North Korean Medical Journal Compared with South Korean Medical Journal (13% North Korea vs. 30% South Korea)

**Figure. 3:**
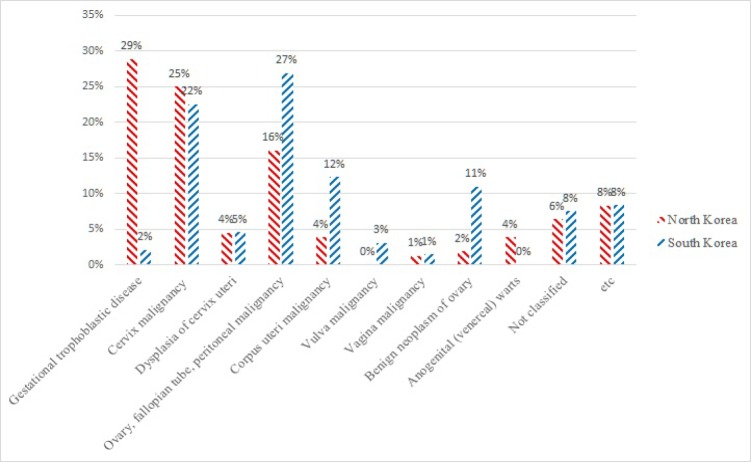
Comparison of Two Korean Medical Journals in Terms of Proportions of Gynecologic Malignancies Classified by ICD-10 between Showed that Proportions of Gestational Trophoblastic Disease, Cervical Malignant Disease, and Anogenital Warts were Higher in North Korean Medical Journal, but Proportions of Ovarian Cancer, Fallopian Tube Cancer, Peritoneal Cancer, Corpus Uterine Cancer, and Vulvar Cancer were Higher in South Korean Medical Journal

In this study, we aimed to infer healthcare status in North Korea with a particular focus on gynecologic oncology using data found in North Korean medical journals. To the best of our knowledge, this was the first report about the current status of gynecologic malignancies in North Korea using recent medical articles conducted in North Korea during last decade.

Furthermore, a comparative analysis was conducted to characterize the different conditions of gynecologic cancer between two Koreas and propose baseline data for doing a large-scale cohort study on the effects of environmental on gynecologic malignancies in the future.

## Materials and Methods

This study was conducted using frequency analysis data obtained from articles on gynecologic malignancies published in South and North Korean medical journals between 2006 and 2016. Frequency analysis is a basic method in research trend investigation limiting the scale of research subject in specific academic field, extracting the corresponding papers, comparing the change of number. Because this study involved the consideration of published literature, the need for a protocol review by the institutional review board was omitted.

*Data Collection*

Nine medical journals were published by the Ministry of Unification’s Information Center in North Korea by the end of 2016 (Kim et al., 2018). We focused our attention on the journal of “*Pediatrics, Obstetrics, and Gynecology*”. This quarterly journal has been established since 1978 . We could find a large number of articles on gynecologic malignancies in this North Korean medical journal.

In order to do a comparative analysis, the Journal of “*Obstetrics and Gynecology Science*” published in South Korea was also considered. This medical journal is published by Korean Society of Obstetrics and Gynecology comprised of an authorized academic group of obstetrician and gynecologist in South Korea. In this journal, we also studied articles which were published from 2006 to 2016. All data such as the names of authors, title, year of publication, and volume were extracted and recorded using Microsoft Excel (Microsoft Corp., Redmond, WA, USA). 

*Data Analysis*

The first framework for analysis were applied to the articles selected for Obstetrics and Gynecology filed. The framework was a classification by main disease of research subject, with reference to the Korean guidelines for subspecialty. In this framework, articles were classified into maternal-fetal medicine, general gynecology, reproductive endocrinology, and gynecologic oncology. Given that the prevalence of breast disease was notable in articles published in North Korean, we added a category named breast disease.

The second framework for analysis is classification of main research subject disease from articles classified as gynecologic oncology by ICD-10. ICD-10 is the 10^th ^revision of the International Statistical Classification of Diseases and Related Health Problems (ICD), a medical classification list by the World Health Organization (WHO). Two researchers checked primary analysis independently. The results from each step were verified mutually. In case of any disagreement, the final decision was made by the corresponding author. Excel was used to calculate the percentages and means. The findings were compared focusing on the differences. The procedures of data collection and analysis are summarized in details in Figure 1. 

## Results

Out of 3361 analyzed articles , 1042 articles were from North Korean medical journal and 1760 articles from South Korea medical journal. There was characteristic lower proportion of articles classified as gynecologic oncology in the North Korean medical journal compared with South Korean medical journal (11% in North Korea vs. 29% in South Korea). Comparison of two Korean medical journals regarding the proportion of subspecialties is provided in Table 1.

From the 116 gynecologic oncologic articles from North Korea, 519 articles from South Korea, main subject diseases were classified by ICD-10. Comparison of proportion of gynecologic malignancies classified by ICD-10 between two Korean medical journals can be seen in Table 2. 

The proportions of gestational trophoblastic disease, cervical cancer, and anogenital warts were higher in North Korean medical journal, but proportions of ovarian cancer, fallopian tube cancer, peritoneal cancer, corpus uterine cancer, and vulvar cancer were higher in South Korean medical journal. 

## Discussion

It is hasty to make a conclusion that specific disease researched frequently in the North Korean medical journal is prevalent actually. However, we can make a conjecture about healthcare status not wholely but partly through the supplement of various grounds involving socioeconomic status. It is difficult to estimate the prevalence of a specific disease, but we can assume that socioeconomic status of North Korea provided the environment making the medical researchers be interested in a specific disease consistently because socioeconomic status can create the grounds for supplying the environment that makes local residents be susceptible to specific disease easily. 

The journal of “*Pediatrics, Obstetrics, and Gynecology*” is the only accessible North Korean medical journal publishing a large number of articles on gynecologic malignancies. This journal is considered to be reputable because there was no change in its publication cycle and dramatic change of amounts of articles during such a long time (since 1978). There is no foreign authors in this journal because it restricts the qualification of paper submission into domestic residence. North Korea is an extremely secluded society and has little foreign intercourse. Academic field is not exception (Ha and Lee, 2018).

We placed emphasis on using objective classification to main subject disease and making reproducible research by another medical researchers. ICD-10 was adopted for the international standard as a warning for the future of global scale cohort study under cooperation with international society.

Given that breast disease is the main subject disease of general surgery in South Korea, we can clear of the breast disease in this research to compare properly with South Korea. In this study, 928 articles from North Korean medical journal and 1,755 articles from South Korea were classified in terms of subspecialty. In terms of subspecialty, We detected lower proportion of gynecologic oncology in North Korean medical journal compared with South Korean medical journal (13% in North Korea vs. 30% in South Korea). Comparison of adjusted proportions of articles classified by subspecialty between two Korean medical journals can be seen in Figure 2.

Following data analysis, we could infer that there was less research interest on gynecologic oncology in North Korea compared with South Korea. In addition, we could make a conjecture about environmental fact affecting such a little interest like poor knowledge of gynecologic oncology due to poor healthcare services provided for gynecologic cancer sufferers. 

While conducting research in North Korea, deep introspection of researcher, especially South Korean researcher is required for analysis of data because retaining objectivity is difficult due to prejudice and paucity of accurate information. Despite such a limitation, the results of this study provided indirect insights into the conditions of the main healthcare issues about gynecologic malignancies in North Korea. Compared with South Korea, research on gynecologic malignancies in North Korea focused on cancer which are more common in developing countries ; whereas, more research in South Korea focused on gynecologic malignancies which were more common in developed countries (Iyoke and Ugwu, 2013). Comparison of proportion of gynecologic malignant diseases classified by ICD-10 between two Korean medical journals suggest such a conjecture in Figure 3.

South Korea can be a model for studying changing patterns of gestational trophoblastic disease. The medical environment in South Korea has improved with developments in socioeconomic status, which has been reflected in changes in incidence of gestational trophoblastic disease. The incidence of H-mole dropped from 40.3 per 1,000 deliveries in 1971-1975 to 2.1 per 1,000 deliveries in 1996-2000. The incidence of gestational trophoblastic tumor dropped from 2.8 per 10,000 deliveries to 0.4 per 10,000 deliveries over the same period (Kim et al., 2004). Such a dramatic disparity of incidence during a long time period in South Korea was similar to disparity of research trends of gestational trophoblastic disease between two Koreas (29% vs 2%). Environmental factors such as socioeconomic gap between two Koreas may influence disparity of research trends of gynecologic cancer between genetic identical two populations.

In addition, we found no article dealing with anogenital wart as a main research subject in South Korean medical journal. On the other hand, 4% of articles in North Korean medical journal investigated anogenital wart. Anogenital warts are very common caused by mostly low risk human papilloma viruses (HPV) (i.e. types 6 and 11). The prevention of anogenital warts can be achieved by the use of the prophylactic HPV vaccine administered prior to sexual debut. The prevalence of female in South Korea decreased after introduction of routine HPV vaccination, principally for females in South Korea (Park et al., 2018b). There is no HPV vaccination in North Korea and such an environmental factor may influence such a difference regarding research trend about anogenital wart between two Korean medical journals.

We found an unique characteristic in the articles published in North Korean medical journal, which is related to alternative medicine. Alternative medicine is the promotion or use of practices which are unproven, disproven, or impossible to prove in an attempt to achieve the healing effect of medicine. We also discovered that a large number of articles in North Korean medical journal were related to alternative medicine; whereas, no article in South Korean medical journal investigated alternative medicine. Previous studies in North Korean medical journal verified the medical effect of traditional medicine compared to conventional medicine. Supporting traditional medicine is the policy of North Korean government and this socioeconomic environment may guide medical researchers to do various studies on traditional medicine (Lim et al., 2009). The other socioeconomic environmental factor may be the shortage of medicines. Shortage of medicines is a fatal problem in health care system of North Korea, which can be worsen by economic sanction (Branigan, 2014). 

The results of this study can be useful to understand the level and focus of medical research in North Korea. There were no articles on laparoscopic surgery for treatment of gynecologic malignancies in North Korean medical journal; whereas, we detected reports on laparoscopic surgery for treatment of gynecologic cancer in South Korean medical journal in the early 1990s (Lee YS, 1994). It is hasty to make a simple conclusion that medical knowledge and technology regarding gynecologic malignancies in North Korea seem to have fallen far behind. Alternatively, we must consider various environmental factors causing such a disparity between two Korean societies.

We also observed a distinctly different research trend between two Korean medical journals concerning gynecologic oncology. Environmental factors such as a socioeconomic gap between two Koreas may contributed to this issue. Grand scale cohort study of environmental effect on gynecologic cancer between two Korean populations in the future will be most interesting medical research field.
